# Multimodal risk assessment of malignancy in thyroidology for nodules with indeterminate cytology

**DOI:** 10.1590/1806-9282.20260055

**Published:** 2026-06-22

**Authors:** Esma Cinar, Demet Sengul, Ilker Sengul

**Affiliations:** 1 Giresun University, Faculty of Medicine, Department of Pathology – Giresun, Turkey.; 2 Giresun University, Faculty of Medicine, Thyroidology Unit – Giresun, Turkey.; 3 Giresun University, Faculty of Medicine, Division of Endocrine Surgery – Giresun, Turkey.; 4 Giresun University, Faculty of Medicine, Department of General Surgery – Giresun, Turkey.

**Keywords:** Thyroid nodule, Biopsy, fine-needle, Ultrasonography, Elasticity imaging techniques, Bethesda system

## Abstract

**OBJECTIVE:**

The aim of this study was to evaluate the clinical, ultrasonographic, and elastographic parameters in the risk stratification of malignancy for thyroid nodules with indeterminate cytology according to The Bethesda System for Reporting Thyroid Cytology.

**METHODS:**

This retrospective, single-center study analyzed 838 thyroid nodules from 716 consecutive patients for six years. The diagnostic performance of nodule size, Doppler ultrasonography features, American Thyroid Association risk of malignancy guidelines, and Tsukuba elasticity scores via strain elastography was assessed. A multinomial logistic regression analysis was employed to compare indeterminate categories (III, IV, and V) with benign cytology (II) to identify independent predictors of malignancy.

**RESULTS:**

Indeterminate nodules exhibited larger mean diameters compared to benign ones (20.83±9.89 vs. 18.65±9.08 mm; p<0.05), and significant associations were identified between III, IV, and V categories and increased peripheral/central vascularization, higher-risk American Thyroid Association risk of malignancy classifications, and elevated Tsukuba elasticity scores (4 and 5). Histopathological malignancy rates were higher in indeterminate groups (p<0.05), whereas patient age and nodule location demonstrated no significant predictive value.

**CONCLUSION:**

Integrated assessment of nodule size, Doppler vascularity, American Thyroid Association risk stratification, and strain elastography significantly enhances the predictive accuracy for malignancy in nodules with indeterminate cytology. These multiparametric indices provide essential guidance for optimizing surgical indications and clinical management in cases of diagnostic uncertainty.

## INTRODUCTION

Thyroid nodules are a common clinical finding, with prevalence rates ranging from 19 to 68% in the general population, largely due to the widespread use of high-resolution ultrasonography (US). While the vast majority of these nodules are benign, a significant proportion necessitate further evaluation to rule out malignancy. Fine-needle aspiration (FNA) cytology, guided by US, is the most accurate and cost-effective method for differentiating benign from malignant thyroid nodules. The Bethesda System for Reporting Thyroid Cytology (TBSRTC) provides a standardized, six-category classification system for FNA results, guiding subsequent clinical management^1–3^.

Among the TBSRTC categories, those classified as indeterminate—specifically, Atypia of Undetermined Significance or Follicular Lesion of Undetermined Significance (AUS/FLUS, Category III), Follicular Neoplasm (FN, Category IV), and Suspicious for Malignancy (SFM, Category V)—present a significant diagnostic and management challenge^4^. The risk of malignancy (ROM) within these indeterminate categories varies widely, typically ranging from 5 to 15% for Category III, 15–30% for Category IV, and 60–75% for Category V^5^. This variability often leads to repeated FNA, molecular testing, or diagnostic surgery, imposing a considerable burden on patients and healthcare systems. Therefore, accurate preoperative risk stratification for these indeterminate nodules is crucial to optimize patient management, minimize unnecessary surgeries, and improve diagnostic precision.

Over the past decade, advancements in imaging techniques and risk stratification systems have aimed to refine the assessment of thyroid nodules. High-resolution US features, such as microcalcifications, irregular margins, taller-than-wide shape, marked hypoechogenicity, and extrathyroidal extension, are well-established indicators of malignancy. However, the subjective nature of US interpretation and the overlap in features between benign and malignant nodules can limit its diagnostic accuracy as a standalone test, particularly in indeterminate cases. To standardize US reporting and improve risk stratification, systems like the American Thyroid Association (ATA) management guidelines and the American College of Radiology Thyroid Imaging Reporting and Data System (ACR TI-RADS) have been developed. These systems integrate various US features to assign a risk score, guiding the need for FNA and subsequent management^6–8^.

Beyond conventional US, emerging technologies such as strain elastography (SE) have shown promise in further characterizing thyroid nodules. SE assesses tissue stiffness, a property often correlated with malignancy, as malignant tissues tend to be stiffer than benign ones^9^. The Tsukuba elasticity score (TES) is a commonly used semi-quantitative method for evaluating nodule stiffness, with higher scores indicating increased stiffness and a greater likelihood of malignancy^10^. The integration of SE with conventional US and clinical parameters has the potential to enhance diagnostic accuracy in indeterminate thyroid nodules, thereby reducing the rate of diagnostic surgeries for benign lesions.

Despite these advancements, the optimal approach to risk-stratifying indeterminate thyroid nodules remains a subject of ongoing research and debate. The present study aims to evaluate the predictive value of various clinical, sonographic, and elastographic parameters—including nodule size, Doppler US (DUS) finding, ATA ROM category, and TES—in differentiating malignant from benign thyroid nodules with indeterminate cytology (TBSRTC, Category III, IV, and V). By retrospectively analyzing a comprehensive dataset from a single center over a 6-year period, we aim to identify robust predictors that can inform clinicians' management decisions for patients with cytologically indeterminate thyroid nodules.

## METHODS

### Study design and patient population

This retrospective cohort study was conducted at a single academic institution, reviewing medical records of patients who underwent thyroid nodule evaluation for 6 years. All patient data were anonymized to ensure confidentiality. A total of 716 consecutive eligible patients possessing 838 thyroid nodules were enrolled in the study. Inclusion criteria comprised patients with thyroid nodules who underwent US-guided FNA and had complete histopathological follow-up or at least 12 months of benign clinical and ultrasonographic follow-up.

### Data collection

For each enrolled patient and thyroid nodule, the following variables were retrospectively collected from electronic medical records: (i) Demographic Data: The relevant age at the time of the FNA procedure, (ii) Nodule Characteristics: Size of the nodule (maximum diameter in millimeters), (iii) Ultrasonographic Features: DUS findings, specifically the presence and pattern of vascularization (DUS 0: no vascularization, DUS I: peripheric vascularization, DUS II: central vascularization, DUS III: peripheric and central vascularization), (iv) The ROM was assessed according to the ATA management guidelines, (v) Elastographic Features: The TES on SE, which was categorized into scores 1–5, with higher scores indicating increased stiffness, (vi) The Cytopathologic Diagnosis: Thyroid nodule cytology was classified according to TBSRTC, and for the purpose of this study, nodules were grouped into indeterminate cytology (TBSRTC III, IV, and V) and benign cytology (TBSRTC II), (vii) The Histopathological Diagnosis: Final histopathological diagnosis, obtained from surgical resection specimens, served as the gold standard for determining malignancy.

### Statistical analysis

The statistical analysis was performed using Statistical Package for the Social Sciences (SPSS) 23.0 (IBM Corp., Armonk, NY, USA). Continuous variables were expressed as mean±standard deviation, and categorical variables as frequencies and percentages. Differences between groups (Category II vs. Category III, IV, and V) were assessed using appropriate statistical tests, including independent samples t-tests for continuous variables and chi-square tests for categorical variables, where applicable. A multinomial logistic regression analysis was employed to assess the independent predictive ability of each variable (age, nodule size, Doppler US findings, ROM on the 2015 ATA management guidelines, and TES on SE) for malignancy in thyroid nodules with indeterminate cytology. The significance level was set at p<0.05. For the multinomial logistic regression, TBSRTC categories were used as the dependent variable, with Category III as the reference category for comparisons, as indicated in the provided statistical output.

## RESULTS

This study retrospectively analyzed data from 716 patients with 838 thyroid nodules. The mean age of the study population was not explicitly stated in the provided summary, but age was not a significantly differentiating factor between the benign and indeterminate groups. Significant differences were observed between the indeterminate cytology and the benign ones across several parameters, including nodule size, DUS findings, ATA ROM categories, and TES.

### Nodule size and age

Thyroid nodules classified as Category III, IV, and V, TBSRTC, exhibited a significantly larger mean size (20.83±9.89 mm) compared to those with Category II (18.65±9.08 mm). The multinomial logistic regression analysis, with TBSRTC as the dependent variable and Category III as the reference category, revealed significant differences in nodule size. Specifically, there was a statistically significant difference in size between Category III and Category I (p<0.10), with Category I nodules generally smaller. A more pronounced significant difference in size was noted between Category III and II (p<0.01), where Category II nodules were smaller than Category III nodules. Age, however, did not demonstrate a significant predictive ability for differentiating between TBSRTC categories in this analysis.

### Doppler ultrasonography

The analysis of DUS findings as an independent variable in the multinomial logistic regression demonstrated its significant predictive value for TBSRTC categories. When DUS was 0 or I, there were significant differences between Category III and I, and between Category III and II. The provided statistical output indicates that specific DUS values are strongly associated with particular TBSRTC categories, highlighting the importance of vascularization patterns in risk stratification.

### Risk of malignancy on the American Thyroid Association management guidelines

The 2015 ATA management guidelines' risk of malignancy (ROMATA) categories also showed significant associations with TBSRTC classifications. The multinomial logistic regression indicated no significant differences between Category II and III groups for ROMATA values of 0, 1, 2, and 3 (p-values: 0.985, 0.981, 0.983, and 0.985, respectively). Conversely, significant differences were observed between Category I and III groups when ROMATA was 0, 1, or 2, with higher ROMATA values generally associated with Category I. No significant differences were found between Category VI and III groups across ROMATA values of 0, 1, 2, and 3.

### Histopathology

Histopathological findings were highly discriminative across all TBSRTC groups. The multinomial logistic regression analysis revealed significant differences across all TBSRTC histopathology groups. Specifically, histopathology values for Category III were higher than those for Categories I and II but lower than those for VI. This indicates that histopathological confirmation remains a critical determinant in the final classification and risk assessment of thyroid nodules.

### Tsukuba elasticity score

The SE, assessed by the TES, also demonstrated predictive utility. The significant difference was observed between Category III and II in terms of TES 1, 2, and 3. In this comparison, Category III nodules exhibited lower TES 1, 2, and 3 values than Category II nodules, suggesting that elastography can help differentiate between indeterminate and benign categories.

### Nodule location

No significant differences were found among TBSRTC groups by nodule location. This suggests that the anatomical position of the thyroid nodule does not significantly influence its classification within the Bethesda system or its ROM in this cohort.

## DISCUSSION

The management of thyroid nodules with indeterminate cytology remains a complex challenge in endocrinology, endocrine surgery, and endocrine pathology. Our single-center, 6-year retrospective study aimed to identify reliable ultrasonographic and elastographic parameters to enhance preoperative risk stratification of these nodules, thereby guiding more appropriate clinical decisions. The findings underscore the significant predictive value of nodule size, Doppler characteristics, ATA ROM categories, and TES in differentiating between various TBSRTC classifications, particularly in distinguishing indeterminate from benign nodules.

Consistent with previous research, our study found that larger nodule size was significantly associated with indeterminate cytology categories compared to benign nodules^11^. This observation aligns with the general understanding that larger nodules may harbor a higher ROM or represent more advanced disease, although some studies suggest that very large nodules might paradoxically have a lower incidence of malignancy^12^. The specific finding that Category I nodules were smaller than III and II provides nuanced insight into the size distribution across TBSRTC categories within our cohort, suggesting a continuum of risk that correlates with increasing nodule dimensions up to a certain point.

**Figure 1:**
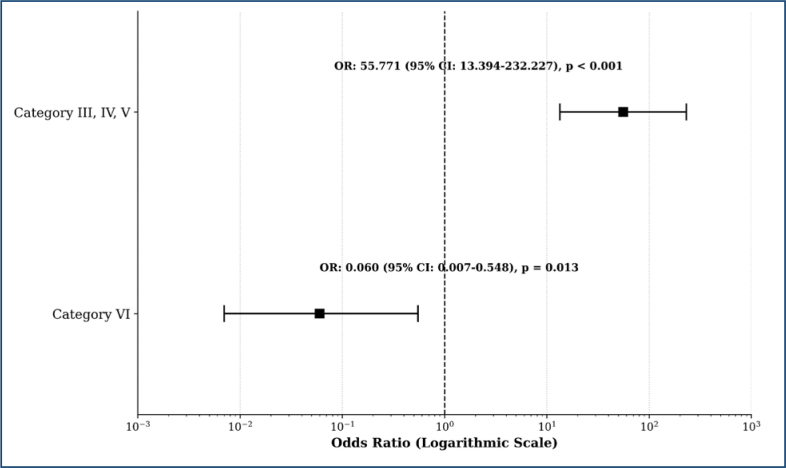
Multinomial logistic regression analysis, evaluating the association between TBSRTC categories and TES (TBSRTC: The Bethesda System for Reporting Thyroid Cytology, TES: Tsukuba elasticity score, OR: Odds Ratio, CI: Confidence Interval).

Doppler sonographic findings, particularly vascularization patterns, emerged as a significant predictor of TBSRTC categories. The presence of central or peripheral vascularization has long been recognized as an important feature for distinguishing benign from malignant thyroid nodules^13^. Malignant nodules often exhibit chaotic or prominent internal vascularity due to increased metabolic demand and neoangiogenesis. Our results, indicating significant differences in TBSRTC categories based on DUS values, reinforce the utility of color DUS as a non-invasive tool to refine risk assessment, especially when integrated with other diagnostic modalities. This is in line with recent literature emphasizing the enhanced diagnostic accuracy when color DUS is combined with other imaging features^14^.

The application of the ROMATA also proved valuable. While no significant differences were observed between Category II and III for various ROMATA scores, significant distinctions were noted between Category I and III, with higher ROMATA values correlating with Category I. This suggests that the ATA guidelines, which incorporate a comprehensive set of US features, are effective in stratifying risk, even within the nuanced landscape of indeterminate cytology. The continuous evolution of such guidelines further highlights the ongoing effort to improve diagnostic precision in thyroid nodule management^15^.

Elastography, specifically the TES, contributed to the differentiation of TBSRTC categories. Our finding that Category III nodules exhibited lower TES 1, 2, and 3 values compared to Category II nodules, thus it might suggest that elastography can provide additional information regarding tissue stiffness. Malignant thyroid nodules are typically stiffer than benign ones, and higher TES scores are generally associated with increased ROM. The specific interpretation of TES values in our results warrants further investigation, as it might reflect a particular stiffness profile within the indeterminate group or an interaction with other nodule characteristics. Nonetheless, the overall utility of elastography in enhancing the diagnostic accuracy of indeterminate thyroid nodules has been consistently demonstrated in systematic reviews and meta-analyses in thyroidology^16–25^.

## CONCLUSION

In essence, this study, while providing valuable insights, is not without limitations. Its retrospective, single-center design may limit the generalizability of the findings. Crucially, histopathological diagnosis remained the ultimate determinant, showing clear distinctions across all TBSRTC groups. This reinforces the role of surgical pathology as the gold standard for definitive diagnosis and underscores the importance of refining preoperative risk stratification to minimize unnecessary surgeries while ensuring timely intervention for thyroidologists in malignant cases.

## Data Availability

The datasets generated and/or analyzed during the current study are available from the corresponding author upon reasonable request.
